# Placebo-controlled dietary intervention of stress-induced neurovegetative disorders with a specific amino acid composition: a pilot-study

**DOI:** 10.1186/s12937-015-0030-3

**Published:** 2015-05-06

**Authors:** Katrin Chaborski, Norman Bitterlich, Birgit Alteheld, Elke Parsi, Christine Metzner

**Affiliations:** 1 Department of Nutrition and Food Sciences, Nutritional Physiology, University of Bonn, Endenicher Allee 11-13, D-53115 Bonn, Germany; 2 Department of Biostatistics, Medicine and Service Ltd, Boettcherstr. 10, D-09117 Chemnitz, Germany; 3 Outpatient Practice of Cardiology/Angiology, Suermondtstr. 13, D-13053 Berlin, Germany; 4 Department of Internal Medicine III, University Hospital, RWTH, Pauwelsstraße 44, D-52074 Aachen, Germany; 5 Bonn Education Association for Dietetics r. A., Fuerst-Pueckler-Str. 44, D-50935 Cologne, Germany

**Keywords:** Psychosocial stress, Hypothalamus-Pituitary-Adrenal (HPA)-axis, Sympathetic nervous system (SN), Amino acid composition, Dietary supplement, Psychological Neurological Questionnaire (PNF)

## Abstract

**Background:**

Psychosocial stress leads to altered neuroendocrine functions, such as serotonergic dysfunction, as well as alterations of the autonomic nervous system and the hypothalamic-pituitary-adrenal (HPA)-axis activity resulting in an imbalance between inhibitory and excitatory neurotransmitters. Poor dietary intake of L-tryptophan as a precursor of serotonin increases sensitivity to stress.

**Methods:**

This randomized, double-blind, placebo-controlled study investigated the effect of a specific amino acid composition with micronutrients on neurovegetative disorders and the cardiometabolic risk profile in psychosocially stressed patients. 32 patients (18–65 years) were eligible for protocol analysis. Points in the Psychological Neurological Questionnaire (PNF), clinical and blood parameter, in particular the serotonin level, salivary cortisol levels, and dietary intake were evaluated at baseline and 12 weeks after supplementation.

**Results:**

The intervention in the form of either verum or placebo resulted in both groups in a significant decrease of neurovegetative symptoms. However, patients of the placebo group achieved significantly less points in the PNF compared to the verum group. But the rate of responders (≥10 points loss in PNF) was not significantly different between the groups. The macronutrient intake did not differ between verum and placebo group. On average, the HPA-axis was not disturbed in both groups. Blood serotonin indicated in both groups no significant correlation with dietary tryptophan intake or PNF.

**Conclusions:**

Daily supplementation of a specific amino acid composition with micronutrients in psychologically stressed patients resulted in no improvement of neurovegetative disorders as measured by the PNF when compared to the placebo group.

**Trial registration:**

Clinical Trials.gov (NCT01425983)

**Electronic supplementary material:**

The online version of this article (doi:10.1186/s12937-015-0030-3) contains supplementary material, which is available to authorized users.

## Background

According to the WHO, psychosocial stress is one of the important health risks of the century; resulting cardiometabolic consequences are enormous [1,2]. Affected persons are usually characterized by neurovegetative disorders as well as metabolic risk factors [1]. Quality of life is reduced by health impairments [2].

Physical or psychological stress leads to an increased secretion of corticotropin-releasing hormone (CRH), which affects two important stress systems: the sympathoadrenal medullary system acts by the hormones epinephrine and norepinephrine on cardiovascular functions, thereby increasing resting heart rate and blood pressure; the enabled hypothalamus-pituitary-adrenal (HPA)-axis goes along with a cortisol secretion [3,4]. Somatic effects of increased cortisol level are mainly displayed in inhibiting immune and inflammatory responses, visceral adipose tissue, reduced insulin sensitivity and rising plasma glucose level [5,6]. Dysfunction of both systems can affect the circadian rhythm of basal cortisol secretion or the cortisol response to a certain stress factor [3], as well as a long-term increase in resting heart rate and blood pressure by hyperactivity of sympathoadrenal medullary system.

The elevated activity of serotonergic neurons in stress diminishes during psychological stress and is associated with mood disorders and depressive symptoms [7]. This is followed by a growing imbalance between the excitatory (norepinephrine, dopamine, glutamate) and inhibitory neurotransmitters (serotonin, gamma-amino-butyric acid [GABA], glycine, taurine). Delivery of amino acids, as precursor of neurotransmitters, within dietary supplements, is an important therapeutic approach and a key feature in nutritional therapy.

The essential amino acid L-tryptophan is the least available amino acid in human nutrition and a precursor of serotonin. In particular, a high protein supply constitutes also the large neutral amino acids (LNAA), which compete with L-tryptophan for the blood brain barrier transporter [8-10]. Central serotonin depletion increases aggressive behaviour and stress-reactivity [11], while poor dietary intake of L-tryptophan also increases sensitivity to stress [12,13]. Therefore, an adequate supply with L-tryptophan during psychological stress is necessary [14]. The same applies to taurine as an inhibitory neurotransmitter and ornithine, which stimulates the release of somatotropin, another lacking factor during stress [15].

The effects of micronutrients on mood, mild psychiatric symptoms and stress were discussed in a recently published meta-analysis [16]. The authors concluded that the recommendations for dietary intake should be reviewed while considering these results. In particular B vitamins [17], and few minerals as iron, selenium, magnesium and zinc are mostly analysed. As an important stress-mineral in the central nervous system [18,19], a hypomagnesemia during stress leads to a pathogenic vicious circle [20].

The aim of this study is to investigate, if daily oral administration of an amino acid mixture in combination with micronutrients, specifically designed to decrease neurovegetative disorders, will target these neuroendocrine and metabolic alterations in adults with psychological stress.

## Methods

### Study population

**Figure 1:**
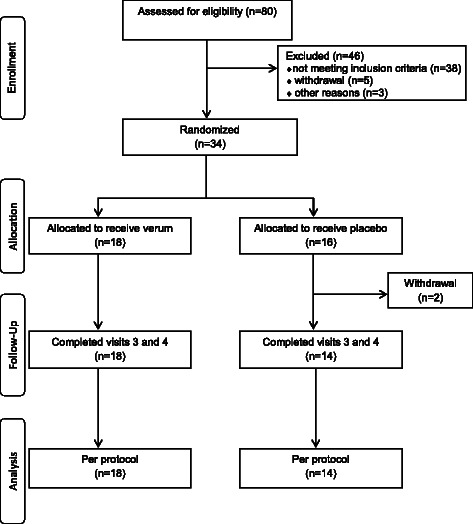
Trial profile.

Psychologically stressed women (n = 21) and men (n = 13) with cardiological disorders were recruited from a cardiological outpatient practice in Berlin, Germany. Psychological stress was defined by neurovegetative disorders determined by a standardized Psychological Neurological Questionnaire (PNF) [21]. Patients aged between 18 and 65 years were included into the study if they obtained 30–50 points in PNF and a resting heart rate ≥ 70/min, considering the antihypertensive drugs. Exclusion criteria were supplementation with dietary supplements or drugs which contain amino acids, vitamins or other micronutrients, therapy with antipsychotic drugs such as tranquilizer or antidepressants, psychological-neurological or psychiatric therapy and acute and chronic diarrhea. The trial profile of the 80 screened participants is shown in Figure 1.

During 12 weeks of the prospective, controlled, double-blind trial, participants were required to take one serving of supplement (8.9 g powder; shown in Table 1) per day solved in 200 ml of water. They got a detailed instruction for consumption of the drink, two hours after the last meal (between 8–10 pm). Compliance was verified by pill counting at week four, eight and twelve.

**Table 1:** **Composition of dietary intervention products**

average values per daily drink (8.9 g powder/0.2 l water)		
	Verum	Placebo
Energy	110.8 kJ/26.9 kcal	146 kJ/34.3 kcal
Protein	3.8 g	>0.0 g
Carbohydrates	0.7 g	7.9 g
Fat	>0.0 g	>0.0 g
**Amino acids**		
L-ornithine	2000 mg	-
Taurine	1000 mg	-
L-tryptophan	800 mg	-
**Vitamins**		
Vitamin C	300 mg	-
Vitamin B6	25 mg	-
Vitamin B2	25 mg	-
Vitamin B1	25 mg	-
Niacin	100 mg	-
Pantothenic acid	100 mg	-
Folic acid	800 μg	-
Vitamin B12	50 μg	-
**Minerals**		
Magnesium	300 mg	-
Zinc	15 mg	-
Selenium	100 μg	-
Chrome	50 μg	-
Molybdenum	50 μg	-

### Psychological neurological questionnaire

Participants answered the PNF at baseline and after 12 weeks of dietary intervention. A borderline range of psychological stress was classified as 30–50 points, assessed at inclusion and baseline. Responders were defined by reaching a loss of 10 points or more from baseline to week 12. The questionnaire comprises a total of 38 items which are divided into five categories of neurovegetative disorders (psychoneurovegetative stability, neurological symptoms, impulsion, arousal, concentration). The self-reported symptoms were assessed as total points, as well as for individual categories, ranging from “not at all” (0 points) to “often” (3 points).

### Clinical and laboratory methods

Prior to dietary intervention and after 12 weeks saliva and blood samples, clinical and biochemical data were collected. After a 10 min resting period, blood pressure and resting heart rate were measured under standardized conditions. The average of three simultaneous measurements was determined. Anthropometric data and venous blood samples were obtained after an overnight fasting period of at least 12 h.

Laboratory Schottdorf MVZ GmbH, Augsburg, Germany, conducted all laboratory analyses (see Additional file 1).

Participants got a detailed instruction for gaining saliva-cortisol samples in the evening (2 hours after last meal, between 8–10 pm) and morning (30 min after awakening) at baseline and week 12. Therefore, Salivette® sterile cotton swabs were chewed for nearly 1 min until saturation and deposited in collection tubes, subsequently stored in a refrigerator. Measurements were performed by using microtiter plate reader Sunrise (Tecan, Crailsheim, Germany) and Cortisol ELISA (IBL, Hamburg, Germany).

Three-day food records were reviewed with each participant at baseline and week 12, the average of 3 days was assessed. PRODI 5.5 software (WVG, Stuttgart, Germany) with database BLS II.3 was used for the analysis.

### Statistical analysis

Statistical comparisons were made between groups using nonparametric Mann–Whitney-*U* test, nonparametric Wilcoxon test was used for data comparison at different time points within groups. Differences in classified variables were tested by Chi^2^ test. All statistical tests were based on per protocol population and two-sided. Differences were considered significant at p < 0.05. Data are reported as mean ± standard deviation (SD). All analyses were conducted using SPSS® for Windows (version 22.0).

The study was approved by the Freiburg Ethics Commission International and was registered with the U.S. National Institutes of Health Clinical Trials.gov (NCT01425983).

## Results

### Patient’s characteristics

A total of 34 patients were randomized into verum and placebo groups, 32 patients were left for protocol analysis (Figure 1). Two patients of the placebo group discontinued the study as early dropouts, both for personal reasons. Daily servings were well tolerated by the majority of patients, one patient of the verum group got a cholecystitis during intervention, two patients of each group indicated gastrointestinal complaints or nausea in the morning. There was no correlation to supplementation assumed.

The patient’s characteristics at baseline are shown in Table 2. The difference of GGT was mainly caused by extreme values in the verum group. About three quarters of patients were overweight. Systolic blood pressure indicated an increased activity of sympathoadrenal system in both groups. But only at inclusion, most patients of verum group met the criteria of elevated resting heart rate (74 ± 10/min), which allowed, just with some limitations, to state an activated sympathoadrenal system. A significant influence on patients with antihypertensive drugs had been excluded.

**Table 2:** **Baseline characteristics of psychologically stressed patients in the verum and the placebo group**

		Verum (n = 18)	Placebo (n = 16)	V vs. P
		$\overline{\mathrm{x}}$ ± SD	$\overline{\mathrm{x}}$ ± SD	*p* -value
sex (n)	f	10 (56%)	11 (69%)	0.429^a^
	m	8 (44%)	5 (31%)	
Age (years)		50 ± 9	50 ± 12	0.670^b^
Height (cm)		172 ± 8	169 ± 10	0.313^b^
Weight (kg)		83 ± 17	82 ± 19	0.772^b^
BMI (kg/m^2^)		28.0 ± 4.4	28.3 ± 4.8	0.986^b^
≤24.5 kg/m^2^		5 (28%)	3 (19%)	0.825^a^
24.6-29.9 kg/m^2^		8 (44%)	8 (50%)	
≥30 kg/m^2^		5 (28%)	5 (31%)	
WC female (cm)		88 ± 11	83 ± 11	0.197^b^
WC f ≥ 88 cm (n)		7 (70%)	3 (27%)	0.050^a^
WC male (cm)		100 ± 11	102 ± 17	0.524^b^
WC m ≥ 102 cm (n)		4 (50%)	3 (60%)	0.725^a^
BP systolic (mmHg)		135 ± 10	131 ± 13	0.175^b^
≥130 mmHgci		14 (78%)	6 (38%)	0.017^a^
BP diastolic (mmHg)		85 ± 10	83 ± 8	0.463^b^
≥85 mmHg		9 (50%)	8 (50%)	1.000^a^
Pulse pressure		51 ± 9	48 ± 10	0.313^b^
rHR (1/min)		69 ± 10	73 ± 10	0.597^b^
PNF (points)		41 ± 5	39 ± 6	0.229^b^
PN (points)		16 ± 3	16 ± 4	0.873^b^
Impulsion (points)		8 ± 2	7 ± 2	0.215^b^
Concentration (points)		7 ± 3	7 ± 3	0.901^b^
Cortisol_m (ng/ml)^c^		7.5 ± 4.4	6.1 ± 3.3	0.552^b^
Cortisol_e (ng/ml)^c^		1.2 ± 1.5	1.2 ± 1.2	0.711^b^
ΔCortisol (ng/ml)^c^		6.3 ± 5.0	5.0 ± 3.8	0.652^b^
Serotonin (μg/l)^c^		180 ± 84	180 ± 47	0.444^b^
CRP sensitive (mg/l)		2.9 ± 3.1	6.6 ± 7.9	0.202^b^
GGT (U/l)		45 ± 48	21 ± 11	0.046^b^
TC (mg/dl)		219 ± 36	217 ± 43	0.772^b^
HDL-C (mg/dl)		59.0 ± 26.8	61.4 ± 18.0	0.356^b^
LDL-C (mg/dl)		133 ± 34	132 ± 42	0.932^b^
LDL-C/HDL-C		2.6 ± 1.0	2.3 ± 0.9	0.330^b^
TG (mg/dl)		162 ± 88	145 ± 57	0.851^b^
TG/HDL-C		3.4 ± 2.6	2.8 ± 1.8	0.621^b^
FPG (mg/dl)		99 ± 28	97 ± 16	0.798^b^
HbA1_c_ (%)		5.8 ± 0.9	5.7 ± 0.3	0.330^b^
HOMA-Index		3.2 ± 3.3	2.0 ± 1.2	0.330^b^
Insulin-ECLIA (μU/ml)		11.9 ± 8.1	8.1 ± 4.1	0.266^b^

abbreviations used: BP, blood pressure; BMI, body mass index; Cortisol: _m, morning; _e, evening; ΔCortisol, cortisol difference morning-evening; CRP sensitive, C-reactive protein sensitive; ECLIA, Enhanced Chemiluminescent Immunoassay; FPG, fasting plasma glucose; GGT, gamma-glutamyltransferase; HbA1_c_, glycated haemoglobin A1_c_; HDL-C, HDL cholesterol; HOMA-Index, Homeostasis Model Assessment-Index; LDL-C, LDL cholesterol; PNF, Psychological Neurological; Questionnaire; PN, psycho-neurovegetative stability; rHR, resting Heart rate; TC, total cholesterol; TG, triglycerides; WC, waist circumference.p-value: ^a^Chi^2^ test; ^b^Mann–Whitney-*U* test; ^c^ data not available for all patients (numbers listed in Additional file 2).

Additionally, the HPA-axis was not disturbed as shown by saliva cortisol measurement and a serotonin deficiency was not detectable in the blood (Table 2 and Additional file 2). Blood serotonin indicated in both groups no significant correlation with dietary tryptophan intake or PNF. Thus, this initial situation was not as expected and complicated the data interpretation.

### Psychological neurological questionnaire

After 12 weeks of supplementation, patients of both groups indicated a significant improvement of neurovegetative disorders. Patients of the placebo group achieved significantly less points in the PNF compared to the verum group (Table 3 and Additional file 2). However, the rate of responders (≥10 points loss in PNF) was not significantly different between verum (n = 7) and placebo group (n = 9; *p* = 0.154).

**Table 3:** **Changing of patient’s characteristics: comparison of values before and after dietary intervention**

	Verum (n = 18)			Placebo (n = 14)			V vs. P
	$\overline{\mathrm{x}}$ ± SD			$\overline{\mathrm{x}}$ ± SD			week 12
	Baseline	Week 12 ^ a ^	Diff	Baseline	Week 12 ^ a ^	Diff	*p* ^ b ^
Weight (kg)	83.4 ± 16.5	84.5 ± 17.1*	1.1 ± 1.7	81.7 ± 19.9	82.2 ± 19.5	0.4 ± 1.2	0.722
BMI (kg/m^2^)	28.0 ± 4.4	28.4 ± 4.6*	0.4 ± 0.5	28.8 ± 4.9	29.0 ± 4.7	0.2 ± 0.4	0.750
WC (cm) f	88 ± 11	89 ± 11	1 ± 2	83 ± 11	84 ± 12	1 ± 2	0.218
Female ≥ 88 cm (%n)^+^	7 (70%)	7 (70%)	0	2 (20%)	4 (40%)	2	0.123
WC (cm) m	100 ± 11	101 ± 11	1 ± 2	109 ± 11	108 ± 12	−1 ± 1	0.368
Male ≥ 102 cm (%n)^+^	4 (50%)	4 (50%)	0	3 (75%)	4 (100%)	1	0.279
BP systolic (mmHg)	135 ± 10	133 ± 13	−2 ± 11	130 ± 14	132 ± 17	2 ± 12	0.955
≥130 mmHg (%n)	14 (78%)	10 (56%)	−4	5 (36%)	10 (71%)	5	0.681
BP diastolic (mmHg)	85 ± 10	85 ± 9	0 ± 8	83 ± 8	84 ± 10	0 ± 10	0.512
Pulse pressure	51 ± 9	50 ± 7	−1 ± 11	46 ± 10	49 ± 11	3 ± 8	0.357
rHr (1/min)	69 ± 10	72 ± 9	3 ± 6	73 ± 10	72 ± 12	−1 ± 9	0.808
PNF	41 ± 5	33 ± 8*	−8 ± 9	39 ± 6	22 ± 9*	−17 ± 12	0.003
PN	16 ± 3	12 ± 4*	−3 ± 3	16 ± 3	10 ± 5*	−7 ± 5	0.168
Cortisol_m (ng/ml)^c^	7.5 ± 4.4	6.6 ± 4.1	−0.5 ± 3.6	6.0 ± 3.4	5.3 ± 3.4	−0.7 ± 3.4	0.425
Cortisol_e (ng/ml)^c^	1.2 ± 1.5	1.5 ± 1.6	0.6 ± 1.7	1.2 ± 1.2	0.9 ± 0.9	−0.3 ± 0.8	0.533
ΔCortisol (ng/ml)^c^	6.3 ± 5.0	5.6 ± 4.8	−0.6 ± 4.1	4.8 ± 3.9	4.4 ± 3.5	−0.4 ± 3.5	0.454
Serotonin (μg/l)^c^	171 ± 90	185 ± 100	5 ± 34	180 ± 50	190 ± 64	11 ± 30	0.356
CRP sensitive (mg/l)	2.9 ± 3.1	2.4 ± 2.6	−0.7 ± 2.9	7.1 ± 8.0	6.0 ± 7.8	−1.0 ± 2.5	0.120
GGT (U/l)	45 ± 48	58 ± 71*	13 ± 25	23 ± 11	24 ± 14	1.6 ± 4.8	0.180
TC (mg/dl)	219 ± 36	211 ± 40	−8 ± 22	217 ± 43	216 ± 36	−1 ± 20	0.561
HDL-C (mg/dl)	59 ± 27	59 ± 26	0 ± 6	62 ± 19	61 ± 14	−0.8 ± 9.3	0.301
LDL-C (mg/dl)	133 ± 34	125 ± 36	−8 ± 19	132 ± 44	132 ± 32	0 ± 21	0.561
LDL-C/HDL-C	2.6 ± 1.0	10.1 ± 32.9	−0.4 ± 0.8	2.3 ± 0.9	2.3 ± 1.0	0 ± 0.5	0.561
TG (mg/dl)	162 ± 88	153 ± 87	−9 ± 39	145 ± 61	136 ± 39	−10 ± 42	0.808
TG/HDL-C	3.4 ± 2.6	6.0 ± 11.9	2.6 ± 11.6	2.9 ± 2.0	2.5 ± 1.2	−0.4 ± 1.1	0.536
FPG (mg/dl)	99 ± 28	102 ± 44	3 ± 17	97 ± 17	92 ± 10	−5 ± 18	0.891
HbA1_c_ (%)	5.8 ± 0.9	5.8 ± 0.9	0.1 ± 0,2	5.7 ± 0.3	5.7 ± 0.3	0 ± 0.3	0.750
HOMA-Index	3.2 ± 3.3	3.6 ± 3.7	0.4 ± 2.6	2.2 ± 1.2	2.5 ± 1.9	0.3 ± 1.8	0.488
Insulin-ECLIA (μU/ml)	11.9 ± 8.1	2.3 ± 1.0	−1.3 ± 6.1	8.8 ± 4.0	10.5 ± 7.5	1.8 ± 6.5	0.955

abbreviations used: BP, blood pressure; BMI, body mass index; Cortisol: _m, morning; _e, evening; ΔCortisol, cortisol difference morning-evening; CRP sensitive, C-reactive protein sensitive; Diff, difference; ECLIA, Enhanced Chemiluminescent Immunoassay; FPG, fasting plasma glucose; GGT, gamma-glutamyltransferase; HbA1_c_, glycated haemoglobin A1_c_; HDL-C, HDL cholesterol; HOMA-Index, Homeostasis Model Assessment-Index; LDL-C, LDL cholesterol; PNF, Psychological Neurological Questionnaire; PN, psycho-neurovegetative stability; rHR, resting heart rate; TC, total cholesterol; TG, triglycerides; WC, waist circumference.

p-value: ^a^Wilcoxon-test within groups, ^b^Mann–Whitney-*U* test: ^*^p < 0.05.

^c^data not available for all patients (numbers listed in Additional file 2).

^+^Verum: women n = 10, men n = 8; Placebo: women n = 10, men n = 4.

### Nutritional impact

Absolute values of energy- and macronutrient-intake did not significantly differ between verum and placebo group (Table 4). Overall, the unfavourable fat-intake based on a poor ratio of n-6 fatty acids/n-3 fatty acids (n-6 FA/n-3 FA) and a large share of saturated fatty acids (SFA). Even after taking the supplements, there was no evidence of a significant change in protein-, fat- or SFA-intake (Table 4).

**Table 4:** **Dietary intake before and after intervention**

	Verum (n = 18) $\overline{\mathrm{x}}$ ± SD		Placebo (n = 14) $\overline{\mathrm{x}}$ ± SD		V vs. P week 12
	Baseline	Week 12 ^ a ^	Baseline	Week 12 ^ a ^	*p* ^ b ^
Energy (kcal)	2627 ± 694	2427 ± 556	2453 ± 625	2601 ± 937	0.779
Carbohydrates (g)	280 ± 88	260 ± 77	248 ± 72	275 ± 114	0.543
Protein (g)	103 ± 23	97 ± 28	101 ± 25	106 ± 33	0.470
Fat (g)	114 ± 40	97 ± 25	107 ± 42	107 ± 41	0.879
SFA (g)	43.1 ± 12.3	37.5 ± 14.0	41.4 ± 15.9	38.0 ± 9.4	0.543
MUFA (g)	41.2 ± 19.5	39.7 ± 19.3	35.7 ± 18.1	35.2 ± 10.8	0.909
PUFA (g)	18.8 ± 9.6	18.3 ± 8.9	15.5 ± 7.5	15.8 ± 5.9	0.543
n-6 FA (g)	16.4 ± 9.1	15.9 ± 8.3	12.7 ± 6.6	13.8 ± 5.7	0.569
n-3 FA (g)	2.0 ± 1.1	2.1 ± 0.9	2.5 ± 2.2	1.7 ± 0.8	0.166

abbreviations used: MUFA, monounsaturated fatty acids; n-3 FA, omega-3 fatty acids; n-6 FA, omega-6 fatty acids; PUFA, polyunsaturated fatty acids; SFA, saturated fatty acids.

p-value: ^a^Wilcoxon-test within groups, ^b^Mann–Whitney-*U* test.

## Discussion

The present study investigated whether daily supplementation of an amino acid mixture with micronutrients specifically decreases neurovegetative disorders in psychologically stressed adults as compared to a placebo. After 12-week dietary intervention both groups demonstrated a significant reduction of characteristic symptoms. Surprisingly the patients of the placebo group reported significantly less points than the patients of verum group.

The PNF could be discussed as a tool for establishing chronic stress [22]. Here, we used a borderline range of psychological stress for determining chronically stressed patients. In order to detect the verum’s effectiveness, pre-post intervention changes in total number of points were analysed, as well as the rate of responder. Considering the results presented here, it seems to be somewhat difficult to distinguish between a placebo effect and the actual efficacy. Two-thirds of the placebo group presented a significantly greater decrease in total points than in the verum group. This placebo effect cannot be explained out of the baseline-situation in comparison to the verum group.

The assumptions of a disturbed HPA-axis and a serotonin deficiency were not met for most patients. Therefore, the diagnosis of psychological stress was merely confirmed by the PNF and in a diminished manner by elevated resting heart rate. Although another clinical study showed that there seemed to be no correlations of blood serotonin and central serotonin in humans [23]. Considering these results, in following studies the blood concentration of amino acids and micronutrients would be even better parameter to control an improvement, especially the relation of available tryptophan to the remaining LNAA [24].

Many unpredictable effects such as changes in nutritional behaviour over time, which are difficult to control for, bias results even in carefully designed studies [25]. The nutritional effect of carbohydrates and protein on mood has been discussed for some time [26-28]. A high-fat diet is supposed to have a protective effect on chronic stress exposure in mice [29]. Therefore, it could be assumed that the composition of consumed food affected mood, behaviour and neurovegetative disorders in the psychologically stressed patients of our study.

Although there were no significant differences in nutritional intake between the groups, the SFA-intake was remarkable. Other authors stated that the impact of a self-medication by eating palatable foods during chronic stress, leads to a high-fat diet in these patients, thereby protecting against stress-induced effects [30,31]. It has also been shown in rodents that a high-fat diet protects against the effects of chronic stress exposure [32,33] while the animals on a low-fat diet reacted with weight gain and increased caloric intake [32]. Psychologically stressed patients often respond with weight gain and an unconscious change of their eating behaviour [34,35]. These changes couldn’t be observed in this study population.

Although considering the protective effects of a high-fat diet in short-term, the increased fat-intake will become habitual [36,37] and thus will lead to a deterioration of the cardiometabolic situation in long-term [38,39]. The after-effects of a long-lasting high-fat diet were shown in rodents and include increasing levels of insulin, insulin resistance and mild hyperglycemia [24,32,40,41].

As a study limitation, there would be to mention the small sample size, which decreases the statistical power, in particular for the comparison of subgroups but also for the detection of marginal changes. The low participation could be attributed to the impacts of chronic stress and should be recognized as a non-participation bias. Concurrently, the selection of participants was locally limited, and most of them were handicapped by cardiological therapy, although significant impairment by antihypertensive drugs was excluded.

The connection between a high-fat diet and chronic stress exposure is widely examined in studies with rodents [29,33]. In this pilot study, we showed that these correlations could also exist in psychologically stressed humans and therefore, provided some clues for further investigations in a larger cohort.

## Conclusions

Daily supplementation of a specific amino acid composition with micronutrients in psychologically stressed patients resulted in no improvement of neurovegetative disorders as measured by the PNF when compared to the placebo group.

## Additional files

Additional file 1:
**Laboratory analyses.**
Additional file 2: **Valid numbers for cortisol and serotonin values.** Considering Tables 2 and 3: ^c^data not available for all patients. Missing cortisol values due to material deficiency for measuring. Missing serotonin values due to value below limit of detection (<16 μg/l).

## Abbreviations

BMI: Body mass index
CRH: Corticotropin-releasing hormone
CRP: C-reactive protein
FA: Fatty acids
GGT: Gamma-glutamyltransferase
HOMA-Index: Homeostasis model assessment index
HPA: Hypothalamic-pituitary-adrenal-axis
LNAA: Large neutral amino acids
n-3 FA: Omega-3 fatty acids
PNF: Psychological Neurological Questionnaire
PUFA: Polyunsaturated fatty acids
SFA: Saturated fatty acids
SD: Standard deviation
SN: Sympathetic nervous system
